# Multimodality Imaging Approaches in Alzheimer's disease. Part II: 1H MR spectroscopy, FDG PET and Amyloid PET

**DOI:** 10.1590/1980-57642015DN94000330

**Published:** 2015

**Authors:** Chetsadaporn Promteangtrong, Marcus Kolber, Priya Ramchandra, Mateen Moghbel, Sina Houshmand, Michael Schöll, Thomas J. Werner, Abass Alavi, Carlos Buchpiguel

**Affiliations:** 1 Department of Radiology, University of Pennsylvania School of Medicine, Philadelphia, Pennsylvania, USA.; 2 Stanford University School of Medicine, Stanford, California.; 3 Karolinska Institutet, Alzheimer Neurobiology Center, Stockholm, Sweden.; 4 Nuclear Medicine Service, Instituto do Cancer do Estado de São Paulo, University of São Paulo, São Paulo, Brazil.; 5 Nuclear Medicine Center, Radiology Institute, University of São Paulo General Hospital , São Paulo, Brazil.

**Keywords:** Alzheimer's disease, dementia, MR spectroscopy, FDG-PET, amyloid imaging, doença de Alzheimer, demencia, espectroscopia, FDG-PET, e imagem amilóide

## Abstract

In this Part II review, as a complement to the Part I published in this supplement, the authors cover the imaging techniques that evaluates the Alzheimer's disease according to the different metabolic and molecular profiles. In this section MR spectroscopy, FDG-PET and amyloid PET are deeply discussed.

## Abstract (pt)

Nesta revisão Parte II, como complemento da revisão Parte I publicada nesta edição, os autores descrevem as técnicas de imagem que avaliam a doença de Alzheimer de acordo com os diferentes perfis metabólicos e moleculares que caracterizam esta doença. Nesta seção são discutidos em profundidade a espectroscopia por ressonância magnética, FDG-PET and imagem com marcadores de peptide beta amilóide.

## INTRODUCTION

More than 5.0 million Americans are currently afflicted by AD. AD affects 5 million people aged more than 65 years and 200,000 individual aged less than 65 years who has younger-onset of AD.^1^ Clinical diagnosis of AD by neuropsychological tests has low reliability, limited sensitivity, and narrow specificity. These tests are most accurate in only the advanced stages of the disease. Advanced neuroimaging modalities pose a challenge for traditional AD diagnosis and monitoring.

Besides neuronal loss, the other hallmark histological changes in AD are the accumulation of abnormal amyloid-β (Aβ) proteins forming the plaques (AP) and neurofibrillary tangles (NFTs).

An ideal neuroimaging marker should be able to accurately detect early neurodegenerative pathology, reflect pathological stages across the entire severity spectrum, predict when an individual with early pathology will become demented, and monitor the effect of a therapeutic intervention on the neurodegenerative pathology.^3^

In this part of the review, the roles and limitations of the biomarkers used in PET and 1H (hydrogen) MR spectroscopy for management of AD are discussed.

## 1H MR SPECTROSCOPY

**Figure 1:**
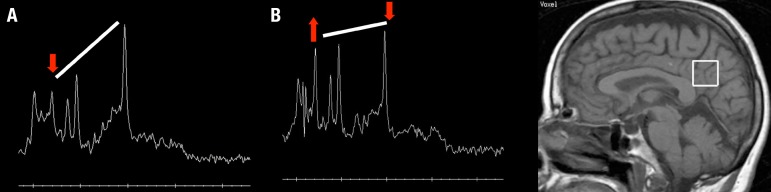
1H MR spectroscopy (1H MRS) in the clinical evaluation of a patient with Alzheimer's disease (AD). The graphic at the top is an example of 1H MRS at the posterior cingulate of a normal volunteer. Below, find an example of 1H MRS of a patient with AD. Note the reduction of N-Acetylaspartate (NAA) and increase of myoinositol (mI) peaks.

Recent date suggest a role of 1H MR spectroscopy (1H MRS) in clinical evaluation of Alzheimer's disease (AD). 1H MRS can detect different metabolic substrates such as N-Acetylaspartate (NAA), creatine and phosphocreatine (Cr) and choline (Cho). Additional metabolites that can be measured with more complex technique are myoinositol (ml), glutamate and glutamine complex (Glx) and lactate (Lac).^1^ The most consistent finding of MRS measurements reported for AD is decreased NAA in many brain regions, which may indicate neuronal loss or mitochondria dysfunction. Subjects with AD has shown reduced NAA in the hippocampus,^2,3^ posterior cingulated,^4,5^ temporal lobe,^6,7^ mesial temporal lobe,^8^ occipital lobe,^6,9,10^ parietal lobe,^6,11,12^ and frontal lobe.^13^ Decrease in NAA of white matter (WM) is observed to be smaller than grey matter (GM) but some authors reported no WM change in NAA.^8^ Other concordant result is increase in mI concentration at several brain locations, which links to gliosis or membrane abnormalities (Figure 1).

The areas involving with increased mI include mesial temporal lobe,^14^ anterior and posterior cingulated,^5,15^ parietal lobe,^11^ occipital^10^ and white matter.^11^ The resonance peak of mI consists of multiple peaks or so called multiplet structures that yield a complex and closely spaced group of resonance lines at clinical field strengths. This broad spectrum pattern is not measured accurately using only single peak of model, which may account for variability in earlier reports. Even recently, despite improvements in automated processing software, clinical group have reported difficulties in obtaining consistent analysis of the mI peak.^1^

Some investigators used ratios between MRS-visible metabolites for distinguish AD from normal subjects. Kantarci et al.^16^ found higher myoinositol/creatinine ratio in the posterior cingulate in AD compared to controls (p<0.001), in AD compared to MCI (p=0.048) and in MCI versus controls (p=0.006). The ratio of NAA /mI was also inferior in AD patients compared to controls (p<0.001), in AD compared to MCI (p=0.002), and in MCI compared to controls (p=0.008). NAA/mI at posterior cingulate provided the highest sensitivity for distinguish AD and control of 82% at the fixed specificity of 80%. Other studies by Wang et al.^17^ found different values in NAA/Cr, mI/Cr and mI/NAA ratios at hippocampus among AD, MCI and normal subjects. However at posterior cingulate, there were different results only in mI/NAA while comparing AD with controls, and AD with MCI. Moreover, they also noted good correlation between mI/NAA and level of cognitive impairment in subjects with AD and MCI.

Conflicting reports about changes of Cho in AD patient has been noted. Some studies report increased Cho.^15,18^ For example, study of Mackey et al.^18^ found elevated Cho/Cr ratio at posterior cingulate and precuneous in AD versus controls. It is suggested that the increase of Cho peak is due to membrane phosphotidylcholine catabolism with the purpose to offer free choline for the insufficient acetylcholine production commonly seen in AD. Cho/Cr decreases with the use of cholinergic agonist drugs in AD which may imply that down regulation of choline acetyltransferase activity may be responsible for the rising of Cho^19^ However, other report no changes^4,6,10,12,20^ or decreases.^5,7^ This discrepancy may be results of differences in protocol MRS or anatomical variation from voxel selection.

NAA/mI or mI/NAA ratios seem to be the most useful parameters due to some reasons. They are independent of Cr values, decreasing variability resulting from age and other factors without having to calculate absolute concentrations. They are also shown to be a dependable diagnostic measure for AD versus controls with high accuracy.^15,16^

Many studies compared the MRS findings in different types of dementia. Schuff et al.^21^ determined the peak values of NAA in subcortical ischemic vascular dementia (SIVD) compared with AD group. SIVD had reduced peak of NAA by 13% in frontal cortex and by 20% in the left parietal cortex as compared with AD subjects. Kattapong et al.^22^ showed lower ratios of NAA/Cr and NAA/Cho in vascular dementia than in AD (p<0.02). Study by Waldman^23^ found higher mI/Cr ratio in AD patients than vascular dementia patients. In contrast with results of Kattapong,^22^ they reported similar findings of NAA/Cr or NAA/Cho between clinical groups. They mentioned that it may reflect the small sample of control subjects and possibly the method of measuring peak heights from spectra, which are scaled to the amplitude of NAA. Ernst et al.^20^ found reduction of NAA and Glx and increasing of mI at frontal lobe in frontotemporal dementia patients while there was no statistically significant frontal abnormality in AD subjects. Some patients in frontotemporal dementia group also showed Lac peak. They reported the overall accuracy for discrimination among group of 84%. Coulthard et al.^24^ reported reduction of NAA/Cr in frontotemporal regions, but not in parietal lobes in frontotemporal dementia. In contrast, study of Garrard et al.^25^ who used MRS to measure metabolites in the posterior cingulate in patients with subtypes of frontotemporal dementia; semantic dementia and progressive nonfluent aphasia subtypes in comparison with AD patients, reported indistinguishable findings between frontotemporal dementia and AD due to overlapped findings of decreased NAA/Cr and increased mI/Cr.

MRS has been studied as a tool to predict which patient with MCI would convert to AD. Modrego et al.^26^ examined 53 patients with aMCI and followed them up for average 3 years, They found by measuring the occipital NAA/Cr ration that MRS could be highly accurate in identifying the true converters. The striking finding was a 100% negative predictive value and an overall accuracy of 88.7%. Interestingly, they found no significant results by doing analysis in the hippocampal and parietal regions. They explained that these inconsistent results with the early involvement of hippocampal and parietal area in AD may be caused by partial volume effects which the large size of the voxel probable included the non-targeted tissue in the analysis or no difference in neuropathological alterations at hippocampus and parietal between converters and non-converters. Longitudinal study by Fayed et al.^27^ recruited 110 subjects with aMCI with a follow up period of 29 months. They reported that MRS measuring the NAA/Cr in the posterior cingulate had sensitivity higher than 80% for predicting who is going to convert to probable AD. However, the distinction of different types of MCY was not possible using MRS.

Godbolt et al.^28^ used MRS in genetic mutated carriers who have a very high risk of developing AD. The investigators demonstrated that NAA/Cr and NAA/mI ratios of carriers were significant lower relative to controls groups. Mean reductions in NAA/Cr and NAA/mI were 10% and 25%, respectively. The reduction of NAA/mI in carriers was related to proximity of expected age at onset.

Correlation between antemortem MRS results and postmortem neuropathology has been studied by Kantarci et al.^29^ The authors found association among decrease in NAA/Cr and increase in mI/Cr, and higher Braak stage, higher neuritic plaque score and more typical histological findings of AD. The NAA/mI proved to be the strongest predictor of the pathologic likelihood of AD. The best correlation noted was that between NAA/mI ratio and Braak stage.

The concordance between MRS and neuropsychological tests are dependent on the type of cognitive deficit the patient presents. Chantal et al.^30^ studied the correlation between medial temporal lobe and verbal memory, parietotemporal lobe and language and visuoconstructional skills, and frontal lobe and executive functions in patients with AD, and found strong correlation between regional MRS changes and the associated-cognitive deficits mentioned above.

The ability of MRS in monitoring effectiveness of therapies in drug trials has studied. Bartha et al.^31^ measured the level of NAA, Cho, NAA/Cr, Cho/Cr, and mI/Cr in non-treated AD patients and followed them after four months of Cholinesterase inhibitor treatment; named donepezil. 1H MRS was acquired at right hippocampus. After treatment it could not be found any cognitive improvement. Decreased level of all the metabolites measured was observed. They concluded that the reduced levels of NAA indicated continued decline in neuronal loss. The decrease in mI level after treatment might indicate a subsequent reduction in reactive gliosis. However, limitations due to small number of subjects and limited time of follow-up should be considered.

**Limitation.** Although recent data suggest that MRS may have a role in clinical diagnosis and prognosis of AD, some limitations have to be discussed. It is important to mention that metabolites ratios provide robust in vivo markers of biochemistry but it has to be interpreted with caution because the ratios are intrinsically ambiguous and prone to misinterpretation.^32^ Technical problems to adjust the TE MRS might contribute to the decrease of the test-retest reproducibility of metabolite measurements. Medial temporal region is one of the most interested site for AD patients. The anterior and mesial portion of the temporal lobe is situated nearby to the tissue-air interface close to the petrous bone. Due to the differences between brain tissue and air magnetic susceptibility, setting a homogenous magnetic field and water suppression within the 1H MRS voxel is complex. MRS can be performed by 2 techniques; single-voxel spectroscopy (SVS) or alternatively, multiple-voxel technique or known as chemical shift imaging (CSI). One of the limitations of SVS is the size of the voxel. Usually it is bigger than the majority of mesial temporal structures, promoting then an effect of partial volume averaging of the adjacent tissue. That also impairs the regional specificity of SVS. 1H MRS at higher Tesla machines would potentially give comparable SNR using smaller voxels. The duration of spectroscopic study is sometimes too long, and that can be a major limitation for less-cooperative AD patients.^33^ Pitfall of MRS could be minimized by applying standard protocols.

## 18F-FDG PET

It has been shown very high diagnostic value of 18F-[2]-fluorodeoxyglucose positron emission tomography (FDG PET) in establishing presence of absence of AD and other neurodegenerative disease with autopsy confirmation. PET is sensitive to change over time, thus, it has value in monitoring disease worsening and therapeutic interventions. FDG PET provides glucose metabolic activity and patients with neurodegenerative dementia show reduced regional cerebral metabolism.

Prodromal AD, a pre-dementia state of mild memory loss while still retaining the ability to perform a daily routine^34^ or MCI due to AD classified as new AD criteria,^35^ may not have the characteristics of more severe AD. However, PET scans performed with FDG show a significant decrease in metabolism in the posterior association cortex, precuneus, and posterior cingulated.^34^ These critical early-diagnostic features may be easily overlooked, as the aforementioned regions generally have a higher glucose metabolic rate than surrounding tissue; impairment would lead them to merely "blend in" to the surrounding regions rather than stand out in a qualitative assessment of an FDG PET scan.^36^ Additionally, patients diagnosed with MCI with AD-like patterns in FDG PET produced scans have been found to eventually develop AD.^34^ These findings demonstrate that FDG PET can potentially be used to predict conversion from MCI to later-stage AD.

Recent study carried out by Shokouhi et al.^37^ proposed a imaging classifier that correlates regional metabolic changes over time, termed regional 18F-FDG time correlation coefficient (rFTC). They have performed a baseline scan and repeated it within an average time of 4.3 ± 1 year. They used linear mixed-effects models to determine different decline rates of rFTC between controls and individuals at risk for AD, then found the association between each subjects' rFTC and cognitive test results. Constant rFTC of controls subjects were found over time whereas in MCI, the values dropped much faster than seen in controls by an additional annual change of -0.02. The decline in rFTC of MCI subjects was also associated with change of cognition. The investigators concluded that this classifier method could be used to monitor cognitive deterioration and disease progression.

Characteristic findings in regions mentioned above highlight the importance of integrating FDG PET more in clinical settings because of its power as an early diagnostic tool. Landau et al. compared the performance of FDG PET with the Functional Activities Questionnaire (FAQ), which is often used to monitor functional abilities in a clinical setting.^38^ It was found that while the FAQ might not catch small changes in a patient's cognitive decline and that FDG measures were strongly associated with a change in FAQ results, illustrating FDG PET's potential to supplement more subjective, clinical forms of diagnosis.

**Figure 2:**
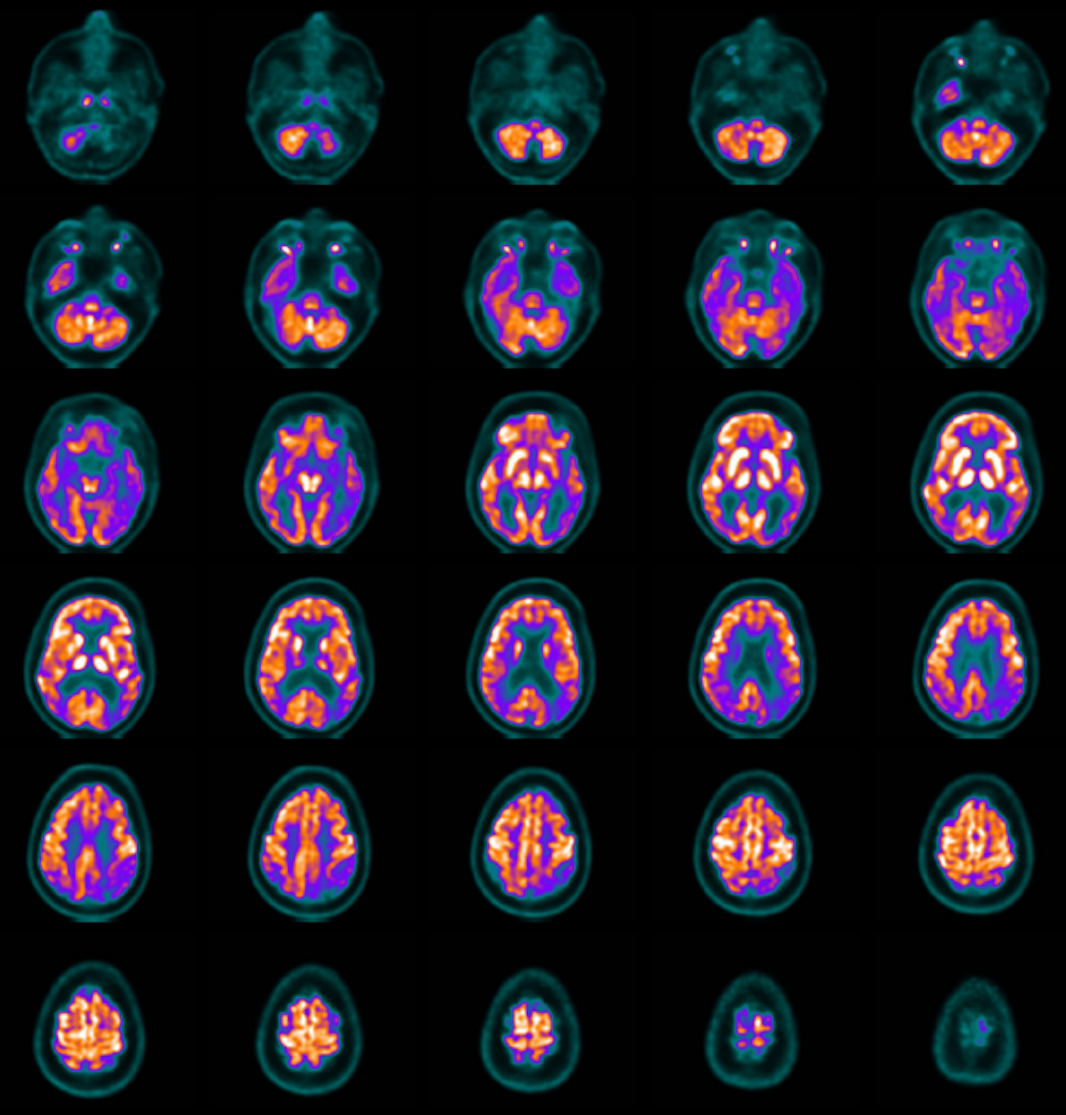
FDG-PET of a patient with Alzheimer's disease. Transversal slices show marked hypometabolism in the posterior cingulate cortex and posterior temporoparietal association cortex. Note the difference in glucose metabolism of the posterior portions of the brain compared to the frontal lobes.

Hallmarks of progressed AD shown by FDG PET include evidence of hypometabolism in posterior regions of the brain, more particularly the temporoparietal region and posterior cingulate (Figure 2).^39^

**Figure 3:**
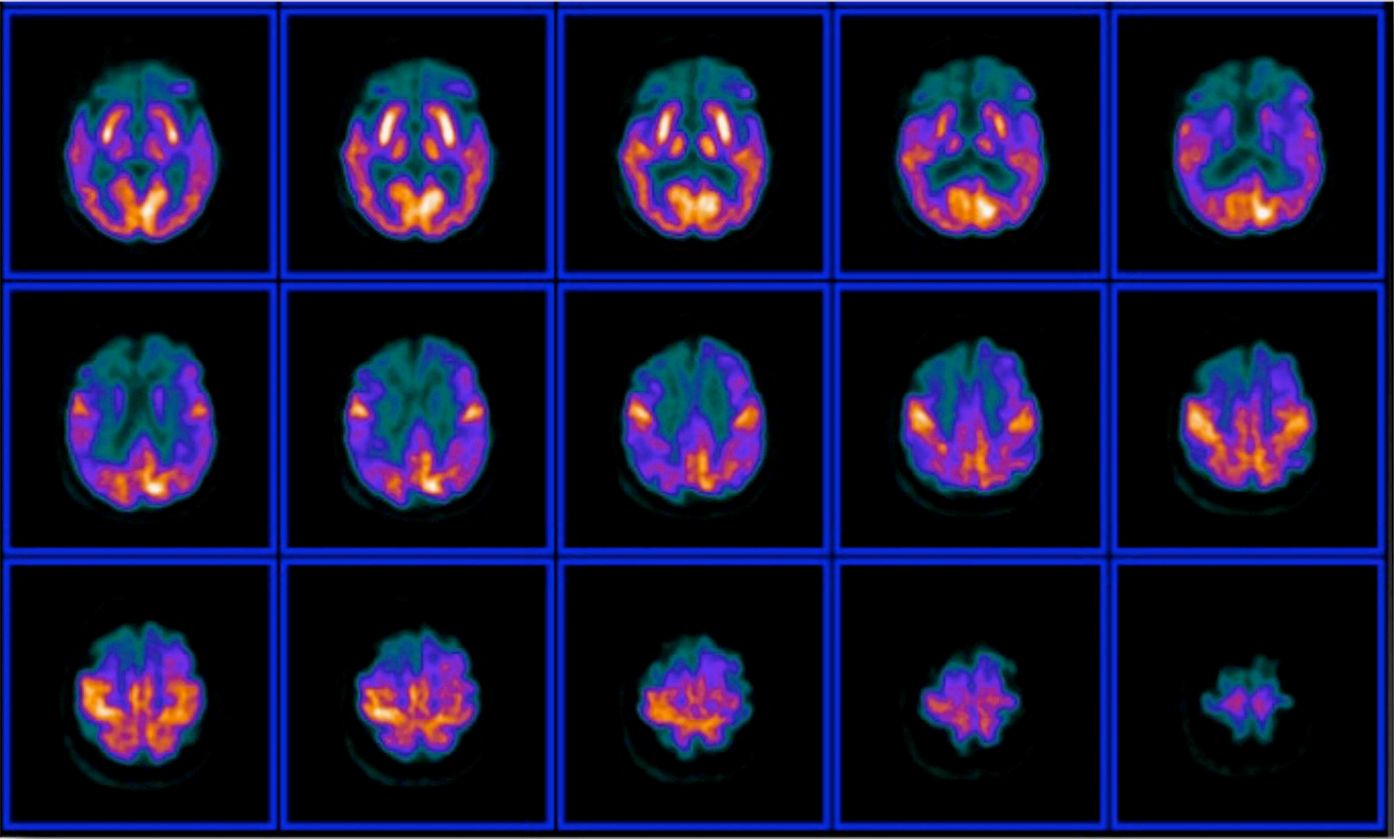
Example of an advanced case of Alzheimer's disease. Note progression of the metabolic posterior temporoparietal impairment towards the frontal lobes, with spared motor and visual cortices activity.

Impairment of the frontal cortex may also be included, but this is associated with later-stage AD and may not occur initially (Figure 3).

Herholz et al. concluded that hemispheric asymmetry might be present, which could be responsible for language and visual impairment.^36^ PET imaging also demonstrates that certain areas of the brain that have been spared impairment in AD, especially the basal ganglia, thalamus, cerebellum, and cortex.^40^ Mosconi et al. suggests that AD-related processes may affect the entorhinal cortex and other regions of the brain, which may facilitate functional impairment.^41^

The initial degree of hypometabolism determined by PET has been shown to correlate with the magnitude of future decline.^40^ Therefore, in addition to showing key characteristics of AD-caused neurological damage, FDG PET has the ability to map the progressive cognitive decline of AD. FDG PET reveals that as AD progresses, parietotemporal hypometabolism becomes increasingly bilateral in addition to the frontal cortex becoming more hypometabolic.^34^ Comorbid conditions can affect the specificity of these predictions. Depressed patients and individuals with abnormal thyroid function have a higher false positive rate for being expected to experience progressing cognitive decline.

**Figure 4:**
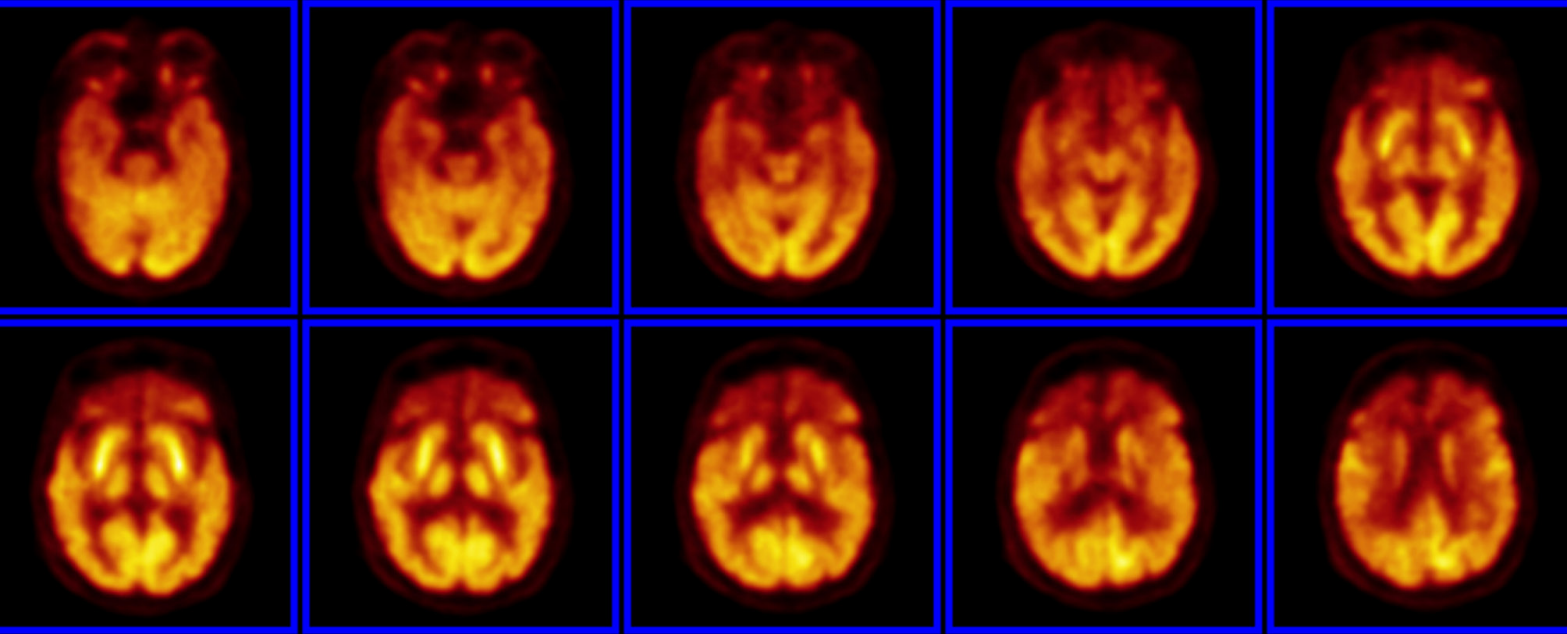
FDG-PET of a patient with a clinical picture of subtle change in behavior and other cognitive impairments suggestive of frontotemporal dementia (FTD). Note the marked reduced glucose uptake in the frontal lobes and in the anterior portion of the temporal lobes. A suggestive PET finding of FTD.

In addition to FDG PET's ability to develop image-based diagnostic criteria for AD, it also has the ability to distinguish AD from similar neurodegenerative conditions. AD and other types of dementia have a characteristic pattern of FDG PET imaging which can be used to differentiate diagnosis in early stage when the specific type remain unclear. FTD, which is often misdiagnosed as AD in its early stages, is characterized by behavioral and language disturbance. Therefore, it could be difficult to distinguish from early AD symptoms in a clinical setting. However, that distinction is easier with FDG PET since reduced regional glucose uptake is seen in frontal and anterior portion of temporal lobes in FTD while that metabolic deficit is seen more in the posterior areas of the brain in AD (Figure 4).

**Figure 5:**
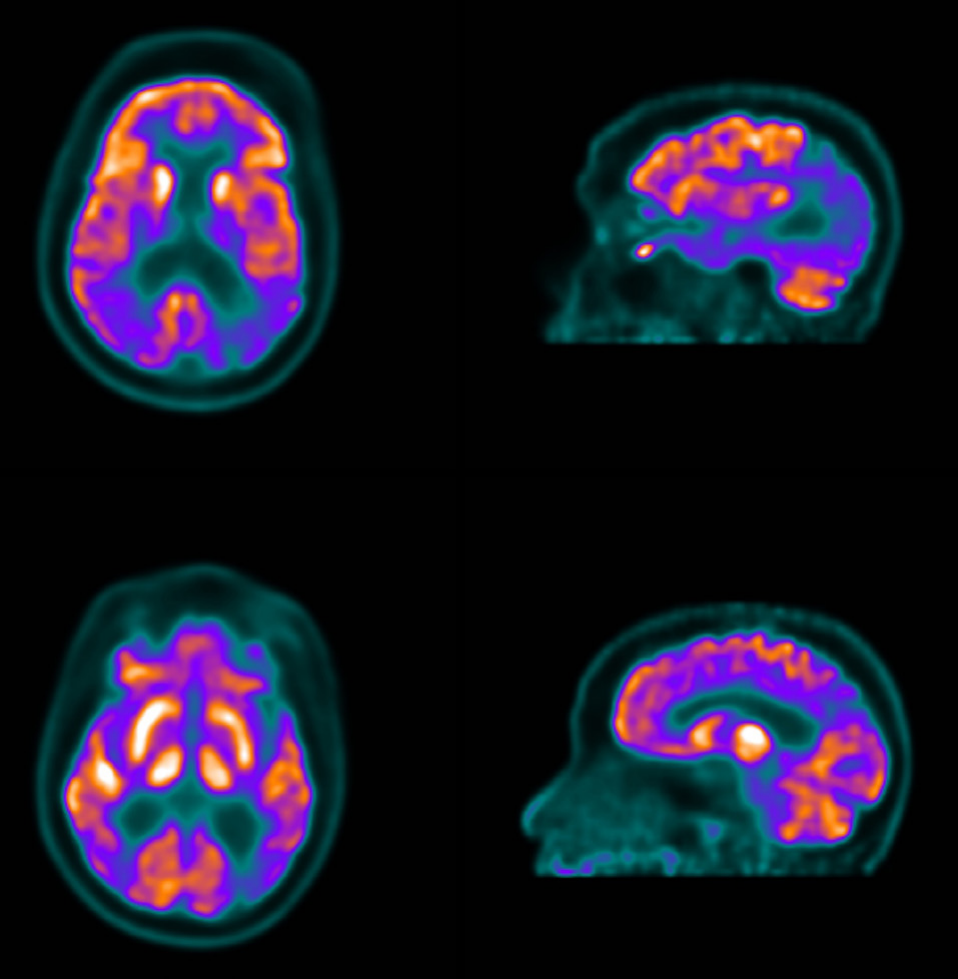
Example of PET findings in Lewy Body Dementia. Note marked hypometabolism in parietal and occipital regions of the brain, a distinct pattern from AD.

Foster et al. showed sensitivity of 97% and specificity of 86% for distinguishing between AD and FTD in the large series of autopsy-confirmed diagnosis.^39^ Other similar conditions, Dementia with Lewy Bodies (DLB), FDG PET shows reduced metabolism in parieto-occipital areas like the primary visual cortex and occipital association areas with normal glucose uptake at association temporal and posterior cingulate cortex, whereas occipital cortex is preserved in AD (Figure 5). In a study of Berti^42^ using postmortem diagnosis, occipital hypometabolic finding can distinguish DLB from AD with 83-90% sensitivity and 80-87% specificity. Other metabolic patterns have been reported in Dementia with Parkinson disease, vascular dementia and Huntington disease.^40^ These findings show that FDG PET is a valuable tool for differential diagnosis between neurological disorders that may appear to be very similar.

Alzheimer's Disease Neuroimaging Initiative (ADNI) and post-mortem studies demonstrated evidence for the power of FDG PET as a biomarker for AD. From reviews literature,^43^ studies that used clinical assessment as the standard provided pooled accuracy of 93%, 96% sensitivity and 90% specificity for distinguishing AD subjects from normal subjects. Silverman et al.^44^ used neuropathological confirmation as the reference standard in testing patients with dementia. Among 138 autopsied subjects, including 97 with confirmed AD, FDG PET yielded the sensitivity of 94% and specificity of 73% for AD diagnosis. FDG PET bears also some prognostic value since it can differentiate a progressive versus nonprogressive course according to the pattern of metabolic changes seen on FDG PET. It showed a negative likelihood ratio of 0.1 (95% confidence interval, 0.06-0.16) from a negative PET scan.

Global disease assessment enhances the accuracy of measurement of FDG PET imaging. The principle of the global metabolic activity is based on multiplying partial volume corrected average SUV to the volume of the organ of interest obtained from anatomical modalities (CT/MRI), the result of multiplying can be named as metabolic volumetric product (MVP). It was first introduced by Alavi et al.^45^ by assessment the brain in AD patients and age-matched controls. They found that by multiplying segmented brain volumes from MRI by mean cerebral metabolic rates for glucose, significant differences between two groups can be demonstrated. This approach requires calculating tissue volume by utilizing modern computer based algorithms and partial volume corrected measurement of metabolic activities at each site of interest. By the same concept, other study by Alavi et al.^46^ investigated 20 patients with probable AD and 17 similar age controls who underwent FDG PET and MRI. They found that atrophy-weighted total brain metabolism (calculated by multiplying the brain volume by the average metabolic rate) showed a very significant difference between two groups (29.96 ± 7.9 for AD and 39.1 ± 7.0 for controls, p<0.001). Absolute whole brain metabolism (calculated by multiplying Atrophy-corrected average CMRglc by brain volume) also showed significant difference which were 37.24 ± 9.65 in AD and 45.09 ± 8.52 in controls, p <0.014). These measurements correlated with mini-mental status exam (MMSE) score. Recent studies carries out by Musiek found that whole brain metabolic volumetrix product (MVP) were significantly lower in AD and accurately distinguished AD patients from controls.^47^

**Limitation.** Some of the limitations include the creation of artifacts and noise during FDG PET image construction, the disadvantages and potential sources of error in both qualitative and quantitative analysis techniques, and disadvantages in semi-quantitative methods. FDG PET imaging can also be affected by pre-existing patient conditions or errors made in protocol during the scanning process.

Another notable limitation, which has been studied extensively, is partial volume error (PVE). Incorrect measurements of tissue activity are due to the limitations of scanners to process structures smaller than 2-3 times the full-width-at-half-maximum spatial resolution of the scanner,^41^ especially in atrophic brain of elderly subjects or AD patients. PVE can also be caused by an incorrect superposition of voxel parameters onto brain tissue causing voxels to contain different tissue types, or tissue fraction. Additionally, patient motion or the movement of either the circulatory or respiratory systems can generate PVE.^48^ Because analysis PET images are dependent on measurements of metabolic activity, and because differential patterns of glucose uptake serve as important characteristics for neurological conditions, it is important that PVE be corrected in order to prevent misdiagnosis or images that show no evidence of abnormality for cases where abnormalities are truly present. Currently, there exist a variety of methods for partial volume correction (PVC), which seeks to curb the problems caused by PVE.

Methods to reduce PVE can include techniques which utilize anatomical information to correct individual voxels, specific regions of interest (multiple or single), or whole images. Other techniques include post-construction methods, using projection data to obtain region of interest (ROI) mean values, or methods that allow for a gradient of activity levels within each region to correct for the assumption that activity within each region is uniform. Techniques to address tissue fraction have also been developed, including methods where edge voxels are treated as multiple tissue types.^48^

Mosconi et al. notes the relative lack of studies used to examine individual cases of MCI, which may be preventing a more detailed understanding of MCI features on an individual level, as well as the dearth of studies which compare MCI to disorders other than AD.^49^

## AMYLOID PET

**Figure 6:**
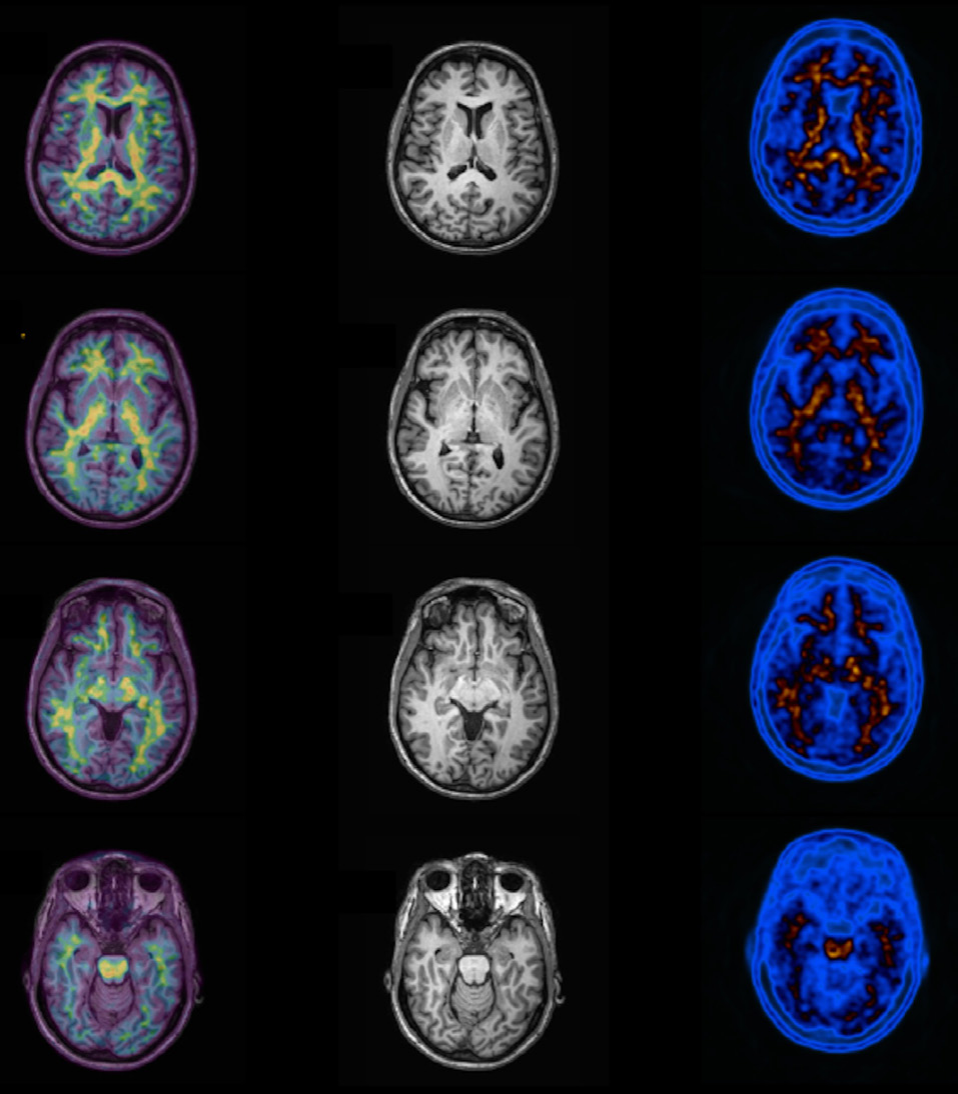
Normal amyloid PET example. Note normal uptake of PIB (Pittsburg Compound) labeled with carbon-11 in white matter tissue at the left column. In the middle column it is shown the MR images of the patient, and in the right column the fused images (PIB + MRI). No cortical uptake of 11C-PIB is seen in this case.

The first amyloid-β (Aβ) PET exam in human was introduced in an individual with probable AD using the 11C-labeled radiopharmaceutical Pittsburgh Compound B (PiB). Amyloid imaging was repeatedly claimed that it is very sensitive technique for the in vivo identification of amyloid plaques into the brain tissue, non invasively, therefore allowing an early confirmation of AD. The normal pattern of amyloid imaging is the white matter deposition of PIB compound, with no cortical uptake (Figure 6).

**Figure 7:**
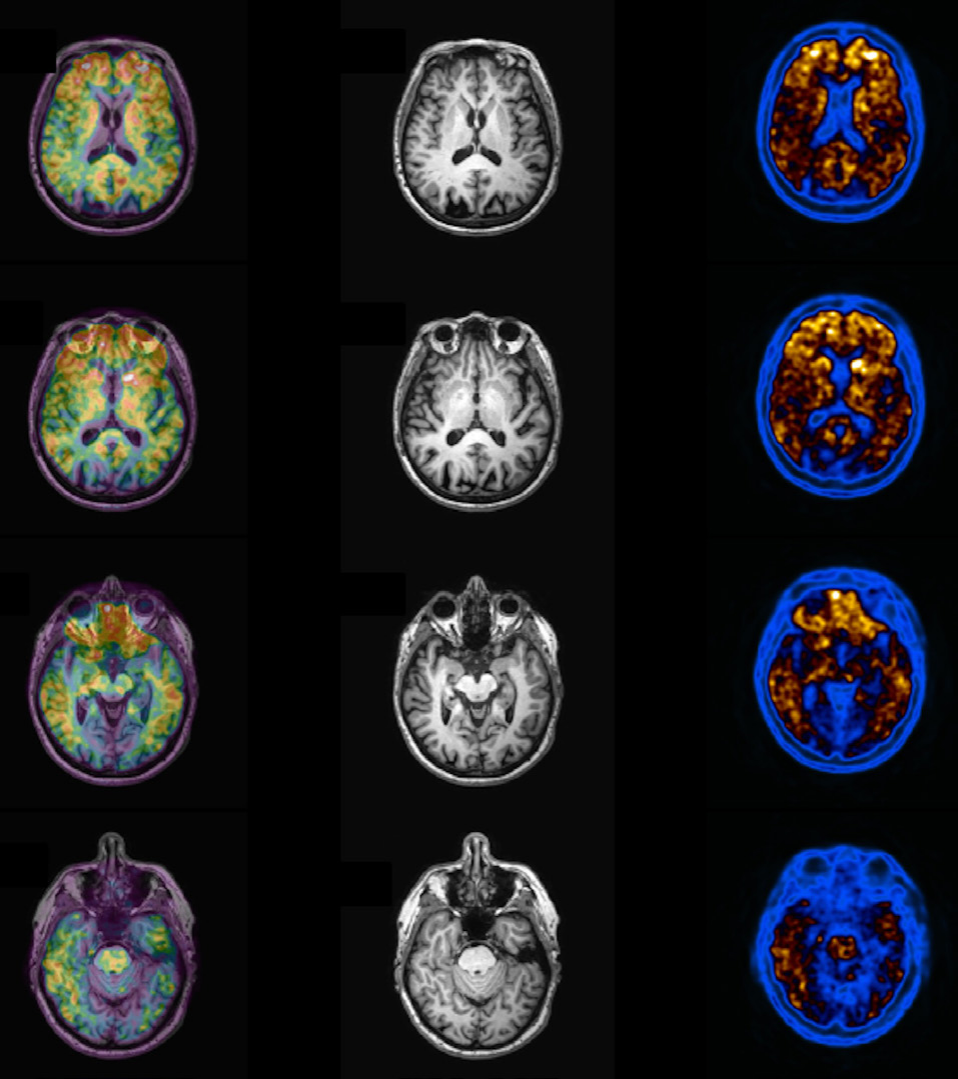
Abnormal PIB PET imaging a patient with confirmed clinical diagnosis of Alzheimer disease. Note marked uptake in the frontal and parietal cortex, with poor visualization of white matter uptake. Right column is the PIB images, middle the MRI and the left column is seen the fused images (PIB + MRI).

Increased cortical PiB uptake in AD compared to controls has been described in many literatures.^50-54^ In AD group, the highest tracer binding is observed at prefrontal cortex, precuneus and posterior cingulate, followed by lateral parietal cortex, temporal cortex and striatum (Figure 7).

The other cortical regions including the hippocampal and amygdala did not show any remarkable PiB uptake compared to controls. Subcortical WM, pons and cerebellum which are unaffected by amyloid deposition showed low PiB binding. At the mean time that PiB was developed, Shoghi-Jadid et al.^55^ used FDDNP labeled with fluorine-18, a hydrophilic radiofluorinated derivative of 2-(1-6-(dimethylamino)-2-naphthylethylidene) malononitrile (DDNP), as a PET tracer to track the deposit sites of neurofibrillary tangles (NFTs) and Aβ senile plaques in the living AD patients. 18F FDDNP have been postulated to recognize amyloid plaque as well as NFTs in living human. Moreover, it is the only imaging agent which visualizes AD pathology in hippocampal region in vivo. 18F FDDNP accumulates significantly in several cortical areas of patients with AD.^56^ Small et al.^57^ reported significantly lower values of FDDNP-PET binding in the whole brain in control group compared to the MCI group as well as lower values in MCI group compared to AD.

Recently three new, longer-lived 18F tracers including 18F florbetapir, 18F florbetaben and 18F flutemetamol have been brought to research and clinical use. In 2012, the Food and Drug Administration (FDA) approved the clinical use of Aβ probe AmyvidTM (18F Florbetapir) for evaluation of patients suspected AD. Clark et al.^58^ used 18F Florbetapir to predict the presence of Aβ in the brain at autopsy. A good correlation was obtained between the visual interpretation of 18F Florbetapir PET imaging and the autopsy findings that confirmed the deposition of Aβ in the brain tissue, according to the standard pathological criteria to define AD. A very high rate of agreement (96%) was seen between amyloid PET imaging and histological confirmation of Aβ. Another study in correlation of 18F Florbetapir and postmortem histopathology was performed by Choi et al.^59^ There was very good correlation of Aβ plaques identified by specific pathological staining techniques, including silver staining and special immunohistochemical assays, and Florbetapir PET imaging pattern. Fleisher et al.^60^ brought 18F Florbetapir PET to clinical cohort of 210 subjects including probable AD, mild MCI and older healthy controls. The data were pooled from four phase I and II clinical trials that used 18F Florbetapir PET imaging under similar protocols. The authors reported that mean (SD) cortical-to-whole-cerebellar SUVRs were significantly distinct among the 3 groups and in the expected direction: 1.39 (0.24) for the probable AD group, 1.17 (0.27) for the MCI group, and 1.05 (0.16) for the controls group (P=2.9x10−14). There also found significant difference of percentage meeting levels of amyloid associated with AD by SUVR criteria (SUVrs greater than or equal to 1.17) and percentage meeting SUVr criteria for the presence of any identifiable Aβ (SUVrs greater than 1.08) among three groups. There was also a strong and direct correlation of florbetapir cortical retention with aging and the presence of APOE ε4 allele (p=0.048).

18F florbetaben has also been shown to bind with Aβ in brain and selectively labeled Aβ plaques and cerebral amyloid angiopathy (CAA) in AD tissue.^61^ Phase II study^62^ proposed sensitivity of 80% and specificity of 91% for discriminating individuals with probable AD form age-matched controls. Phase III studies in 238 patients from 17 centers have reached completed.^63^ The investigators claimed 100% sensitivity and 92% specificity of 18F florbetaben PET at subject level analysis but 77% sensitivity and 94% specificity for regional detected Aβ as compare with postmortem diagnosis. Ong et al.^64^ found high Aβ burden in 53% of MCI subjects when using SUVr 1.45 as a threshold. There is a good direct correlation between 18F florbetaben and PiB global SUBr values with almost same diagnostic power to differentiate AD from healthy subjects.^65^

18F flutemetamol PET with visual assessment has been reported 93.1% sensitivity and 93.3% specificity against standard of truth among AD, MCI and healthy controls subjects.^66^ Duara et al.^67^ suggested an additive information from 18F flutemetamol PET and sMRI in classifying amnestic MCI subjects. The overall correct classification rate for amnestic MCI from 18F flutemetamol PET using SUVr 1.4 and medial temporal atrophy derived from sMRI was 86%. Longitudinal study^68^ in AD and amnestic MCI with 2-year follow-up reported 18F flutemetamol PET SUVr showed clear group clustering while hippocampal volume showed extensive overlap between group. A longitudinal study showed that more that 89% of the converters came from the positive flutemetamol group. Pooled results of phase III studies in 18F flutemetamol have not been announced yet.

Johnson et al.^69^ reviewed recent publications in clinical dementia setting and reported 96% of AD patients were amyloid positive. On the other hand, amyloid-negative scans in patients with the diagnosis of probable AD would represent imprecise clinical diagnosis or that patients bear very small amount of tissue amyloid plaques that PET could not detect, and by following them up it will be detected years ahead.

Although a number of new PET probes are currently under investigation in academia and under development by pharma companies, there are some concerns with respect to the clinical value of Aβ imaging and questions have been recently raised. Moghbel et al.^70^ reviewed the technical aspects and described several potential problems, such as partial volume effects resulting in underestimated SUV data, high ratio of nonspecific to specific WM uptake and discordance between the concentration of Aβ in the brain with histopathological and immunohistochemical studies and question about the specificity of these tracers. Investigators in amyloid imaging field have answered some Moghbel's questions,^71^ however, some issues still need to be clarified. Kepe et al.^72^ proposed the lack of in vivo binding validation of these probes and the consequent deficiency in the understanding of their tissue binding and specificity. It is uncertain how amyloid agents interact with many form of Aβ. Lockhart et al.^73^ demonstrated that PiB clearly delineated classical plaque as well as diffuse plaque and CCA. It was also found to label NFTs with lower intensity than Aβ pathology. Cairns et al.^74^ reported case diagnosed mild AD whose PiB PET showed unremarkable but positive biofluid markers. However, autopsy performed 2.5 years after scan showed lesions that met neurofibrillary stage III and Braak and Braak stage C. There was no evidence of any other neurodegenerative or clinically meaningful vascular disease. Aβ deposition is also an important pathology in Downs's syndrome. In addition, Aβ has been reported as an additional pathology in Parkinson's disease, dementia with Lewy Bodies, Pick's disease, corticobasal degeneration, amyotrophic lateral sclerosis and progressive supranuclear palsy.^75^ Ly et al.^76^ found nearly most ischemic stroke patients in their study has a high PiB uptake within the peri-infarct region compared to the contralateral side, particularly in the WM around the infarct region. The cause of the focal PiB retention was uncertain and requires further investigation. There are also evidence that suggests even cognitively-normal patients may have high levels of 11C-PIB, ligand used to detect Aβ, suggesting that a large degree of Aβ buildup may not always translate into the development of AD symptoms. Healthy elderly controls can also show high PIB retention.^77,78^ Some PIB positive elderly healthy controls have demonstrated normal cognition.^79^ Moreover, it is common to see numerous degenerative changes including NFTs and Aβ plaque in a large number of cognitively normal individuals.^80^

Additionally, the rapid peripheral and central metabolism of these probes and the brain transport of metabolites are severe limitations at the very heart of the tracer design and development. These limitations cause extensive and nonspecific uptake of amyloid agents in WM which affects both AD patients and controls.^72^ Many studies found non-negligible WM uptake in both AD and controls.^65,66,81^ Recent study by Barthel^62^ reported the highest 18F florbetaben SUVr in cerebral WM as compared to other cortical and subcortical regions. Nonspecific WM uptake can produce spillover and partial volume effect into neighboring GM which should be concerned in atrophic AD brain. The extensive WM uptake can make unreliable imaging interpretation. Moreover, this phenomenon provide additional evidence that PiB and stilbene derivatives are nonspecific to Aβ target as some studies showed these probes can bind to myelin with high affinity.^82-84^ Villemagne et al.^71^ have addressed that this WM uptake is similar between AD and normal controls and that partial volume effect is not an exclusive limitation to amyloid PET imaging but affects equally all PET image procedures. They claimed even by knowing that this limitation had not proven to be a major obstacle to the quantitative analysis of Aβ deposits in cortical GM, visual assessment was of higher priority than absolute quantification and localization for many clinical purposes. However, the authors accepted that a lot of improvements must be accomplished regarding the development of more sensitive and specific probes, with lesser WM concentration, and that will allow the incorporation of more suitable imaging tools to quantify and better classify patients with cognitive impairment.

In present days, it well recognized that Aβ deposition starts in the preclinical AD, increases up to the time when the AD diagnosis is confirmed clinically, and then remains under a plateau as disease progresses. Cerebral amyloidosis itself is not sufficient to promote cognitive deficits in AD which is more related with FDG PET and sMRI as the biomarker of neurodegeneration. Anti- Aβ therapies have been repeatedly reported to be ineffective. Thus, there is no validated clinical value of amyloid imaging in monitoring disease progression.

Considering the limitations discussed above, the amyloid imaging demands careful discussions in the proper clinical utility. Recently, the Society of Nuclear Medicine and Molecular Imaging (SNMMI) and the Alzheimer's Association (AA) have developed the appropriate use criteria for amyloid PET.^85^ It is suitable for individuals with stable or progressive unexplained MCI, satisfying core clinical presentation either an atypical clinical course or an etiologically mixed presentation and progressive dementia, and atypically early age of onset. Patients with one of these appropriate criteria should have the following characteristics:

1) a cognitive deficit confirmed by an objective neuropsychological test;
2) A diagnosis of possible AD, but when the diagnosis is uncertain after a comprehensive evaluation by a dementia expert; and
3) when the recognition of the pathological status of Aβ is expected to increase diagnostic certainty and change management. The inappropriate situations include patient that fulfill the diagnostic criteria for probable AD under typical age of onset, to determine the level of cognitive impairment, based solely on a positive family history of dementia or APOE ε4 presentation, unconfirmed clinical examination of cognitive impairment, suspected autosomal mutation carriers, asymptomatic individuals and nonmedical use such as legal, insurance coverage, or employment screening.

However, there is a lot of skepticism regarding the value of amyloid imaging to significantly change outcomes and management of patients with prodromal and even AD. The main issue is that Aβ PET findings are not specific to AD and about 30% of older people have Aβ and do not have AD and will not have AD.^86^ Then in July 2013, the Centers for Medicare and Medicaid Services (CMS) released a draft decision memo indicating that Medicare would pay for contrast-enhanced PET scans aimed at visualizing beta-amyloid protein plaques in patients brains only in the contest of rigorous clinical trials, under the agency's "coverage with evidence development" (CED) policy. The decision mainly focuses on the role of positive scan, while the guideline of SNMMI and AA considers both on positive and negative findings which negative finding would rule out an AD. CMS reported that use of the scans to exclude AD in narrowly defined and clinically difficult differential diagnoses is promising. Nevertheless, CMS acknowledged that more evidences need to be discovered, including when the scan would replace or complement other biomarker for particular patient subpopulations.

**Limitation.** Amyloid imaging tracers do not meet the fundamental advantage of PET that is different from other imaging modalities as the ability of quantitative functional assessment of specific tissue in human. Appropriate amyloid PET probe should provide the signal only from Aβ retention and its peripheral metabolites should be minimize or pass blood-brain-barrier that can be predicted for quantification. For recent evidence, the in vivo specificity of the amyloid agents has not been fully established and the sources of non-specific uptake have not been identified. Moreover, the technical limitation in PET system has not been corrected.

Even though all limitations are not considered, the diagnostic value of amyloid imaging is still questionable. Current criteria for the neuropathological diagnosis of AD by National Institutes of Aging-Alzheimer's Association^87^ uses 3 parameters including (A) immunohistochemistry-derived Aβ plaque score described by Thal et al.,^88^ (B) NFTs stage from immunohistochemistry for tau or phosphor-tau, and (C) neuritic plaque score from Thioflavin S or modified Bielschowsky as recommended by Consortium to Establish a Registry for Alzheimer's disease (CERAD) protocol to obtain "ABC" score and transform into one of four levels of AD neuropathologic change: Not, Low, Intermediate or High. For Aβ plaque score, other method that identifies progressive accumulation of Aβ deposition in medial temporal lobe only is recommended as it is highly correlated with Thal phases.^88^ Present status of amyloid imaging may provide information of neuritic plaque that fulfills only criteria (C), however, it cannot yield appropriate signal in the medial temporal lobe and insensitive to tau deposition. Thus, amyloid imaging shows no enough strong evidence that it is suitable for AD diagnosis which is the most indication that described in literatures.
